# Effect of water scarcity during thermal-humidity exposure on the mineral footprint of sheep

**DOI:** 10.5713/ajas.20.0350

**Published:** 2020-07-14

**Authors:** Jalil Ghassemi Nejad, Bae-Hun Lee, Ji-Yung Kim, Kyu-Hyun Park, Won-Seob Kim, Kyung-Il Sung, Hong-Gu Lee

**Affiliations:** 1 Department of Animal Science and Technology, Sanghuh College of Life Sciences, Konkuk University, Seoul 05029, Korea; 2 Team of an Educational Program for Specialists in Global Animal Science, Brain Korea 21 Plus Project, Sanghuh College of Life Sciences, Konkuk University, Seoul 05029, Korea; 3 National Institute of Animal Science, RDA, Cheonan 31000, Korea; 4 College of Animal Life Sciences, Kangwon National University, Chuncheon 24341, Korea

**Keywords:** Ewes, Minerals, Thermal-humidity Exposure, Water Scarcity, Wool

## Abstract

**Objective:**

Combination of two stressors on alteration of mineral footprints in animals needs due attention to meet maximum production and welfare, particularly in grazing sheep. This study tested whether ewes (*Ovis aries*) exposed to water deprivation and thermal–humidity stressors had altered mineral footprints in their wool, serum, urine, and feces.

**Methods:**

Nine ewes (age = 3 years; mean body weight = 41±3.5 kg) were divided among a control group with free access to water, and treatment groups with water deprivation lasting either 2 h (2hWD) or 3 h (3hWD) after feeding. Using a 3×3 Latin square design, animals were assigned to treatment groups for three sampling periods of 21 days each (n = 9). Blood was collected by jugular venipuncture. Wool was collected at the end of periods 2 and 3. Metabolic crates designed with metal grated floors were used for urine and feces collection. We measured sodium (Na), magnesium (Mg), phosphorus (P), chloride (Cl), calcium (Ca), manganese (Mn), copper (Cu), iron (Fe), and zinc (Zn).

**Results:**

The wool mineral levels did not differ between the treatment groups, although K was marginally lower (p = 0.10) in the 2hWD group. The serum and urine mineral levels did not differ between the treatments (p>0.05). Fecal K was significantly lower in the 2hWD group than in the other groups (p≤0.05).

**Conclusion:**

In conclusion, water deprivation and thermal-humidity exposure altered the excretion of K, but not of other minerals, in the wool, urine, feces, or serum of ewes. Thus, no additional mineral supplementation is needed for water deprived ewes during thermal-humidity exposure.

## INTRODUCTION

Minerals leave the transport pool by secretion (e.g., milk, sweat, and digestive juices) and excretion (e.g., urine and feces), both of which may intensify in hot environmental conditions [1]. Sheep (*Ovis aries*) often search for water while grazing under thermal-humidity exposure (THE) and may excrete more minerals than normal through sweat, urine, feces, and wool. In addition, different species of livestock and physiological periods may have different mineral requirements. For instance, grazing cows require more Mg during lactation [2]. Growing young and reproductive females also requires higher mineral levels than other physiological stages [3]. This phenomenon may impact mineral requirements [4,5] and act as an environmental pollutants [6–8]. Furthermore, it is well documented that mineral supplementation can regulate oxidative stress from growth or external stressors such as a hot climate [1,9]. On the other hand, pasture plants including legumes that contain more Ca, P, Mg, Cu, Zn, and Co and usually less Na and Mn than grasses may be altered during extreme environmental conditions [2,9]. In addition, maturity, climatic and seasonal changes can influence forage minerals [10]. Thus, the mineral supply provided by pastures is not adequate for grazing ruminants during certain parts of the year. Mineral excretion should be traced via body matrices and can be used as an index of mineral requirements, particularly during water shortages and THE.

Mineral regulation in the body depends on internal factors, including bioavailability, mineral reserves, age, physiological status, and excretion rate, and external factors such as environmental conditions [11]. Minerals are regulated through intestinal absorption, excretion, and endogenous losses such as biliary, pancreatic, or intestinal secretions [5]. In this regard, homeostatic mechanisms help regulate mineral levels. The mineral footprint can be measured in biological matrices such as blood, hair, urine, and feces. The concentrations of Ca, P, and Fe in hair have been shown to reflect the mineral status of cattle [12]. The mineral levels in wool and other matrices reflect the mineral concentrations in the body and in blood and are useful for monitoring nutrition status. Thus, the mineral levels in hair, blood, urine, and feces can be used as an index to estimate animal requirements, mineral deficiencies, or over supplementation [1]. Yet, there is a paucity of information regarding the nutritional background of minerals in animals.

We tested whether water deprivation and THE alter the macro- or micro-mineral levels in ewes. We measured Na, Mg, P, Cl, Ca, Mn, Cu, Fe, and Zn in wool, serum, urine, and feces.

## MATERIALS AND METHODS

### Animal housing and experimental design

The experimental methods were approved by the animal welfare and ethics authority of Kangwon National University, Chuncheon, Republic of Korea (KIACUC-16-0098). Non-pregnant, non-milking Corriedale ewes (n = 9; age = 3 years; mean body weight = 43±3.5 kg) were individually housed in metabolic crates (0.75×1.45 m) with metal grated floors designed for urine and feces collection. Steel pads with small holes under the cages were used to collect feces and urine. The ewes were acclimated to the experimental conditions for 10 days before the start of the experiment. Individuals were assigned to one of three treatment groups according to a Latin square design (3×3) for three periods of 21 days each. Each 21-day period consisted of an acclimation period (14 days) during which sheep adjusted to the new treatment group, and a measurement period (7 days) during which samples were collected. The treatment groups included free access to water (FAW), 2 h water deprivation (2hWD), and 3 h water deprivation (3hWD) following feeding. Following the water restriction, ewes were given free access to water.

Feed was weighed and provided as a commercial total mixed ration (TMR; Table 1) twice daily at 09:00 and 18:00. Ewes were fed based on maintenance requirements throughout the experiment, so that no residual feed remained. The average dry matter (DM) intake was 728±82.0 (g/d). Commercial mineral blocks (Red Rockie, Dae Dong Bio Co. Ltd., Daegu, Korea) were provided in feeding buckets to avoid mineral deficiency; their contents included Na (38%), P (1.0%), Ca (1.0%), Mg (0.5%), Cu (300 mg/kg), I (150 mg/kg), Co (50 mg/kg), Mn (200 mg/kg), Zn (300 mg/kg), Se (mg/kg), vitamin A (60,000 IU/kg), and vitamin D_3_ (6,000 IU/kg). Water was provided in buckets and was available *ad libitum* for the FAW group. Water was provided at 11:00 and 20:00 for the 2hWD group, and at 12:00 and 21:00 for the 3hWD group; thus, ewes in the 2hWD and 3hWD groups were provided water at 2 and 3 h post-feeding, respectively.

### Sample collection

Feed samples were taken at the beginning and end of each 7-day sampling period, pooled for each period, dried, and stored for further analyses. Urine (g and mL) and feces (g) were collected daily during the last 7 days of each period and stored for analysis. Fecal subsamples (~200 g/d) were taken from each sheep and placed in a plastic bag, dried at 65°C for 3 days, finely ground (1 mm), and analyzed for nutrients via ether extraction and the dry ash method [13]. Blood was collected via jugular venipuncture into two evacuated tubes without additives (BD, Franklin Lakes, NJ, USA) at 13:00 on day 21 of each period. After collection in tubes, serum was obtained by centrifugation (1,200×g for 20 min) and then stored at −20°C until the mineral analyses.

### Temperature-humidity exposure analysis using temperature-humidity index

Sheep were housed in a climate-controlled environment where temperature and humidity were monitored hourly (Table 2) throughout the trial using a data logger device (CEM-DT-172, No. 11048007; CEM, Shenzhen, China). The temperature–humidity index (THI) was calculated using the following equation from Marai et al [14]:

THI=db °C-[(0.31-0.31 RH)(db °C-14.4)]

Where, db °C is the dry bulb temperature (°C) and RH is the relative humidity. The THI values were interpreted as ≤22.2 = absence of heat stress, 22.2–23.2 = moderate heat stress, 23.3–25.5 = severe heat stress, and ≥25.6 = extremely severe heat stress [14]. Based on Marai et al [14] and a previous study from our laboratory [15], this calculation suggests that the sheep in this experiment were extremely heat-stressed, as the average THI was 29.1 throughout the experiment (Table 2).

### Wool sampling and preparation

At the end of each period (21 days), wool was carefully shaved from the neck at 13:00 using commercially available pet grooming clippers (Hair Clippers, Model 7200; Rikei Trading Co., Ltd., Seoul, Korea) [15,16]. The wool samples from the 2nd and 3rd period were only used for the mineral profile analysis since the only re-grown wool could be used as an index for the mineral accumulation in the shaft. The wool samples were numbered, placed into dry polypropylene tubes (conical tube; HM Hyundai Micro Co., Seoul, Korea), stored in a plastic bag, transported to the laboratory, and stored at room temperature as described by Ghassemi Nejad et al [17] before pre-treatment.

For pre-treatment, 0.1 g of wool was weighed and placed in a 50 mL polypropylene tube (DigiTube; SCP Science, Baie-D’Urfe, QC, Canada) with 5 mL of 65% to 70% nitric acid. After gently mixing, the tubes were incubated on a heating block at 80°C for 1 h, during which time each tube was gently mixed every 20 min. The tubes were then removed and cooled to room temperature. Distilled water was added to bring each sample volume to 50 mL. Elements with high concentrations were further diluted with 5% nitric acid for analysis.

### Mineral analysis

Calibration standards were prepared by appropriately mixing 100 ppm of multi-element standard (Mg, Mn, Fe, Cu, and Zn; SCP Science, Canada) and 1,000 ppm of single element standards (Na, K, P, and Ca; SCP Science, Canada) and serially diluting with 5% nitric acid. For Cl calibration, NaCl (Sigma, St. Louis, MO, USA) was dissolved in pure water and diluted in 5% nitric acid.

Minerals and heavy metals were measured using an inductively coupled plasma mass spectrometer (Agilent 7700X; Agilent Technology, Santa Clara, CA, USA). Element concentrations (ppm) were calculated using the standard curves prepared with certified mass spectrometry standards (SCP Science, Canada) after adjusting the sample dilution factor. Similar procedures were used to measure the mineral levels in serum, urine, fecal, and feed samples.

### Statistical analysis

Statistical analyses were performed using SAS (version 9.0; SAS institute Inc., Cary, NC, USA). We used a generalized linear model that included the effects of individuals, treatment group, and sampling period as follows:

$${\text{Y}}_{\text{ijkl}}=\mu +\alpha_{\text{i}}+{\text{S}}_{\text{j}}+{\text{P}}_{\text{k}}+{ɛ}_{\text{ijkl}}$$

Where Y_ijkl_ = each observation, μ = total mean, α_i_ = effect of treatment group, S_j_ = effect of individual identification, P_k_ = effect of sampling period, and ɛ_ijkl_ = error. The effects of individual and sampling period were not significant. A Duncan’s multiple range test was used to rank group means with a significant F-test. Differences were considered statistically significant at p≤0.05, and differences with 0.05≤p ≤0.10 were accepted as marginally significant. The THI data were averaged for weekly means (on hourly intervals) and analyzed by repeated measures analysis of variance. The covariance structures (autoregressive order 1, unstructured, and compound symmetry) for the repeated measures model were tested and the structure that fit the model best was chosen.

## RESULTS

In the present study, we obtained mineral profiles, including Na, Mg, P, Cl, K, Ca, Mn, Fe, Cu, and Zn, for wool, urine, serum, and feces. No differences were observed between the treatment groups in the mineral levels of wool, including Na, Mg, P, Cl, K, Ca, Mn, Fe, Cu, and Zn, during sampling periods 2 and 3 (Table 3; p>0.05). Since we only used the re-grown wool shaft to represent mineral accumulation, the wool from 2nd and the 3rd cut was used. The urine (Table 4) and serum (Table 5) mineral levels, including Na, Mg, P, Cl, K, Ca, Mn, Fe, Cu, and Zn, did not differ among the three groups (p>0.05). In feces (Table 6), K concentration was lower in the 2hWD group than the FAW and 3hWD groups (p≤0.05). No other minerals, including Na, Mg, P, Cl, Ca, Mn, Fe, Cu, and Zn, differed in fecal samples between the groups (p>0.05).

## DISCUSSION

Minerals are essential for animal health and immunity, and mineral deficiencies can reduce disease resistance [2,18]. Grazing animals such as sheep receive their minerals from pasture forage and grasses that their mineral contents can be altered due to rainy seasons, climatic factors, seasonal changes and hot/cold stress [10]. Additionally, the overconsumption of some essential minerals, and their interaction with other minerals, may be toxic [19,20]. Sheep are the most susceptible ruminant species to chronic Cu toxicity. When assessing dietary requirements, it is essential to consider mineral surpluses as well as mineral deficiencies, as both can negatively impact animal health [21]. For example, Cu uptake is inhibited by Mo, S, and Fe in ruminants, whereas high levels of Ca in feed inhibit Zn uptake [1]. Higher Cu and Zn levels are expected in the presence of environmental stressors such as THE and drought [1,9]. Even though the risk of mineral toxicity is low, excess minerals may be excreted and act as environmental pollutants [6–8]. Absorption efficiency of many minerals that affect their bioavailability are influenced by association of minerals with fiber fractions in feedstuffs and or binding of minerals to undigested fiber fraction. Accordingly, we previously indicated that water deprivation and thermal stress can alter fiber digestibility in sheep [16].

Wool is an indicator of long-term mineral accumulation yet showed no difference between sampling times or water deprivation under THE in this study. This result suggests that 3 h without water in ewes under THE does not alter mineral accumulation in sheep wool. Sheep are less susceptible to drought and THE than other ruminants and can adapt to these conditions [22]. Collecting blood, urine, or feces for analysis is usually impractical in light of restricted resources and trained workers. Hair and wool can be easily collected and stored at room temperature for analysis. Furthermore, hormones such as cortisol [15] accumulate in hair as it grows and can be a good index for possible long-term measures. We did not find changes in the wool mineral contents of sheep, despite THE and 2 and 3 h water shortages; this suggests that these stressors do not dysregulate minerals in the body. Homeostatic mechanisms may mitigate the effects of water shortages and THE during prolonged periods, as reported in another study of ewes [22]. However, whether longer durations of water shortage can affect mineral levels during THE remains unknown and requires further study.

While wool accumulates minerals as it grows over long time periods, urine can be used as a daily indicator of mineral status [12,15]. In the present study, we found no difference in the urine levels of Na, Mg, P, Cl, K, Ca, Mn, Fe, Cu, and Zn. A previous study indicated that free access to water increased urine output in ewes compared with 2 and 3 h of water deprivation [23]. The water deprivation in this study can alter urine output [23], but changes in urine volume and urination frequency had no effect on mineral levels. This suggests that the mechanisms for mineral regulation maintain homeostasis even in the presence of stressors.

Serum minerals are considered quick-responding nutrient biomarkers. Changes in external factors, including daily feed or environmental stressors, can be observed in the blood of the affected animals. A survey conducted to quantify serum mineral concentrations in Montana ram lamb populations indicated that approximately 9.5% of ranch flocks were deficient and 57.1% were marginally deficient in Zn [24]. This can apply to other mineral profiles as well. Diets that are marginally low in Cu are a risk factor for infectious disease, but excess Cu increases the risk of sudden death. In addition, some minerals such as Se, Cu, Zn [25], and Fe [26], the main co-factors in antioxidation, are pivotal to enhance the immune system and prevent oxidative stress. Moreover, the roles of minerals in growth [27,28], feeding and digestion [18,29], and reproduction [30,31] in ruminants are well established, but this research has mainly been done on cattle. In sheep, it has been reported that adding water to TMR during THE and water deprivation does not change serum biochemical and hematologic parameters or circulating hormone levels [16,22]. Fan et al [2] stated that among other minerals, Na and P deficiencies could be prevalent in sheep. The fact that the serum mineral levels did not show any changes in this study suggests that THE and water deprivation were not effective stressors. Future studies that use longer deprivation times may produce different results.

Analyzing feces can provide insight into mineral regulation during the previous days or weeks. However, in the present study we did not find any differences in fecal mineral levels, except for K, between the treatment groups. K is the 3rd most abundant mineral in many animals (following Ca and P), and the first sign of its deficiency is often reduced feeding. Under low K intake, other minerals exhibit deficiencies in equilibrium with K. The K-deficient rats also had lowered K and increased Na in their muscle tissue, whereas control rats had the opposite pattern [5]. Sato and Dobson [32] showed that 24 h following a single intradermal injection of aldosterone, Na^+^ concentrations in sweat decreased by 15%. Thus, the body can adjust to K deficiency. Moreover, the kidneys have a key role in K and Na regulation through urine and feces [6]. K-deficient rats showed an approximate equilibrium among these elements, and its elimination appeared to be normal. K is absorbed almost completely from the gastrointestinal tract and is excreted in urine with various acid radicals [6], with low but fairly consistent excretion of K in feces. By contrast, our data showed inconsistent fecal K excretion, possibly because water deprivation resulted in drier feces and thus a lower K level. Water scarcity during THE may also confound mineral excretion due to homeostasis and changes in excretion rate, as found in previous studies [15,16,22,23]. However, we found no differences in the mineral levels of feces, except for K; the reason why fecal K was higher in FAW and 3hWD compared with 2hWD remains unknown. If the ewes were fed *ad libitum* and pasture grazed, the higher K levels in pastures and free access to water could have resulted in a higher DM intake and K excretion; however, this was not the case as the ewes were given commercial feed.

Heart and kidneys have relatively low K storage [6]. Research by our laboratory found that sheep adapt to heat and water deprivation [22]. Water restriction increases blood cortisol concentrations in sheep [33], whereas lactating cows are more sensitive to water restriction [14,34,35]. This suggests that stress levels are exacerbated by longer water restriction.

Overall, no differences in mineral levels were found in wool, serum, or urine between the FAW, 2hWD, and 3hWD groups, suggesting that the ewes receiving 2 or 3 h of water deprivation did not need increased mineral supplementation. Moreover, 3 h of water deprivation during THE did not increase mineral excretion (except for K) in wool, serum, urine, or feces in this study. This implies that water deprivation up to 3 h during THE does not alter mineral levels.

## CONCLUSION

Water deprivation for up to 3 h under THE altered the excretion of K via wool and feces, but not other minerals, in wool, urine, feces, and serum of ewes. This implies that no additional mineral supplementation is needed for water deprived ewes during THE. Therefore, wool and feces would be the most prominent methods of choice for similar studies. Our initial hypotheses that water scarcity together with thermal stress would increase mineral excretion via body matrices was not confirmed in this study. However, whether a longer duration of water shortage can affect wool mineral levels during THE remains unclear and requires further study. More research is needed to understand the decreased K levels in the 2hWD group.

## Figures and Tables

**Table 1:** Ingredients and chemical composition of the total mixed ration (n = 6)

Items	Levels	Minerals (ppm)	Mineral levels
	
Ingredients (g/kg)		Na	949.5±19.9
Crushed corn	39.9	Mg	2,991.5±75.4
Formula feed	49.9	P	6,125.2±728.4
Corn flakes	44.9	Cl	14,783.7±3,545.7
Wheat flakes	139.7	K	11,061.6±125.4
Wheat bran	49.9	Ca	7,199.9±1,030.8
Corn gluten	49.9	Mn	121.2±2.2
Sesame oil meal	25.0	Fe	339.3±17.2
Brewers grain	194.6	Cu	24.2±0.9
Liquid yeast	172.0	Zn	90.8±2.3
Tall fescue	62.4		
Ryegrass	74.9		
Mixed hay1)	62.4		
Molasses	20.0		
Vitamin-mineral premix2)	9.5		
Chemical composition (g/kg)			
Dry matter	600.0		

1) Mixed hay included: alfalfa 18.7 g/kg, Bermuda grass 12.5 g/kg, timothy hay 6.2 g/kg, oat hay 9.4 g/kg, ryegrass 12.5 g/kg, limestone 3.1 g/kg.

2) Contained (per kg of DM): 10,250 IU of vitamin A, 2,050 IU of vitamin D, 20 mg of vitamin E, 10 mg of CuSO_4_5H_2_O.

**Table 2:** Average morning, noon, and evening temperatures and humidity patterns in the experimental house (07:00–20:00)

Item	Hours	Values	SEM
Temperature	07:00–08:00	28.7	0.1
	12:00–13:00	32.3	0.2
	19:00–20:00	29.1	0.1
Relative humidity	07:00–08:00	71.3	0.5
	12:00–13:00	85.9	0.9
	19:00–20:00	90.2	1.1
THI1)	07:00–08:00	27.5	-
	12:00–13:00	31.5	-
	19:00–20:00	28.2	-

SEM, standard error of the mean.

1) The temperature-humidity index was calculated as suggested by Marai et al [14].

**Table 3:** Average wool mineral levels in ewes with water deprivation under temperature-humidity exposure (n = 9)

Minerals (ppm)	Grouping1) (1st sampling)			p-value	Grouping1) (2nd sampling)			p-value
	
	FAW	2hWD	3hWD		FAW	2hWD	3hWD	
Na	1,465.5±5.3	1,306.0±122.0	1,211.5±160.2	0.187	1,242.9±126.3	1,162.9±66.3	1,279.5±206.6	0.625
Mg	72.7±9.1	78.5±11.1	62.0±6.6	0.174	63.7±8.5	73.4±13.2	72.6±11.2	0.534
P	202.3±68.9	146.5±17.2	157.3±3.5	0.285	149.4±9.2	140.9±11.7	154.6±3.3	0.344
Cl	9,408.0±448.2	9,434.0±304.6	10,123.0±763.9	0.833	8,540.9±660.9	8,547.0±267.4	9,009.3±585.7	0.839
K	20,715.0±1,466.9	21,550.0±1,752.4	22,831.0±1,139.4	0.347	23,958.0±2,205.3	19,898.0±2,331.5	20,356.0±1,584.4	0.099
Ca	462.6±78.7	449.6±27.8	402.6±25.7	0.557	360.6±40.0	409.5±36.2	484.9±65.3	0.154
Mn	2.2±0.4	2.2±0.4	1.7±0.2	0.249	2.3±0.4	1.9±0.6	1.6±0.9	0.755
Fe	250.7±76.8	229.8±38.5	238.0±56.4	0.991	348.6±135.5	219.7±68.9	130.4±22.9	0.135
Cu	5.8±0.9	4.4±0.9	4.8±0.4	0.202	5.1±0.9	5.2±0.5	5.3±1.1	0.966
Zn	159.4±33.5	135.0±14.5	135.3±16.7	0.793	143.1±4.7	150.7±15.0	141.5±14.6	0.948

1) FAW, free access to water; 2hWD, 2 h of water deprivation following feeding; 3hWD, 3 h of water deprivation following feeding.

**Table 4:** Average urine mineral levels in ewes with water deprivation under temperature-humidity exposure (n = 9)

Minerals (ppm)	Grouping1)			p-value

	FAW	2hWD	3hWD	
Na	6,110.7±1,881.8	5,817.5±1,766.2	5,400.9±1,519.3	0.715
Mg	226.3±81.3	182.8±63.3	235.6±83.3	0.413
P	24.4±7.8	17.1±5.6	39.1±21.4	0.143
Cl	5,002.1±1,078.0	5,288.4±1,448.6	5,247.7±1,197.3	0.871
K	1,746.0±663.3	1,663.7±329.9	2,120.5±766.1	0.304
Ca	182.8±87.5	200.6±87.5	207.0±64.2	0.815
Mn	0.2±0.9	0.1±0.05	0.2±0.09	0.261
Fe	3.2±1.1	2.9±0.8	3.0±1.5	0.867
Cu	0.6±0.1	0.7±0.07	0.6±0.1	0.458
Zn	3.0±0.3	2.9±0.3	3.1±0.3	0.247

1) FAW, free access to water; 2hWD, 2 h of water deprivation following feeding; 3hWD, 3 h of water deprivation following feeding.

**Table 5:** Average serum mineral levels in ewes with water deprivation under temperature-humidity exposure (n = 9)

Minerals (ppm)	Grouping1)			p-value

	FAW	2hWD	3hWD	
Na	3,442.6±55.0	3,474.2±104.3	3,523.6±158.6	0.576
Mg	24.6±3.4	25.4±1.9	24.7±2.5	0.847
P	115.2±16.1	122.2±13.1	117.0±8.9	0.688
Cl	3,749.3±185.7	3,792.6±98.8	4,077.6±394.8	0.130
K	313.7±34.4	292.1±22.1	273.8±35.4	0.270
Ca	141.2±25.1	140.3±21.6	133.8±7.7	0.880
Mn	0.01±0.004	0.01±0.004	0.01±0.007	0.748
Fe	2.3±0.4	2.0±0.4	2.1±0.1	0.596
Cu	1.1±0.05	1.2±0.1	1.2±0.1	0.934
Zn	3.1±0.3	3.4±0.5	3.4±0.2	0.322

1) FAW, free access to water; 2hWD, 2 h of water deprivation following feeding; 3hWD, 3 h of water deprivation following feeding.

**Table 6:** Average fecal mineral levels of ewes with water deprivation under temperature-humidity exposure (n = 9)

Minerals (ppm)	Grouping1)			p-value

	FAW	2hWD	3hWD	
Na	5,930.1±1,520.4	6,321.0±687.7	6,660.9±496.3	0.793
Mg	4,730.6±471.2	5,127.6±395.1	5,071.6±195.5	0.422
P	13,451.9±1,123.1	12,188.6±950.4	14,145.1±1,030.4	0.474
Cl	14,623.6±1,762.9	13,634.6±1,443.2	12,347.6±1,579.5	0.848
K	7,169.6±894.9^a^	4,571.8±1,425.2^b^	5,111.9±896.4^ab^	0.050
Ca	15,779.9±801.2	15,456.3±602.1	15,879.1±1,126.1	0.828
Mn	365.6±27.5	408.8±46.7	381.7±36.2	0.420
Fe	4,244.6±386.1	4,049.9±346.5	3,467.6±1,191.0	0.783
Cu	77.0±8.9	89.9±17.2	80.8±12.2	0.511
Zn	372.1±32.5	403.8±79.4	373.5±75.3	0.807

1) FAW, free access to water, 2hWD, 2 h of water deprivation following feeding; 3hWD, 3 h of water deprivation following feeding.

a,b Means within a row followed by a different letter are significantly different (p≤0.05).
